# Examining the disparities: A cross-sectional study of socio-economic factors and food insecurity in Togo

**DOI:** 10.1371/journal.pone.0294527

**Published:** 2023-11-27

**Authors:** Komlan Kota, Marie-Hélène Chomienne, Sanni Yaya

**Affiliations:** 1 Interdisciplinary School of Health Sciences, Faculty of Health Sciences, University of Ottawa, Ontario, Canada; 2 Department of Family Medicine, Faculty of Medicine, University of Ottawa, Ottawa, Canada; 3 School of International Development and Global Studies, University of Ottawa, Ottawa, Ontario; 4 The George Institute for Global Health, Imperial College London, London, United Kingdom; National Autonomous University of Mexico, MEXICO

## Abstract

**Background:**

Despite many interventions, Togo continues to have one of the highest rates of poverty and food insecurity in the sub-Saharan African region. Currently there is no systematic analysis of the factors associated with household food-insecurity in this country. This study aimed at exploring the factors associated with food insecurity in Togo.

**Methods:**

This was a cross-sectional study that used data from five waves (2014 to 2018) of the Gallup World Poll (GWP) for Togo. Sample size included 4754 participants, aged 15 and above. Food insecurity was measured using the Food Insecurity Experience Scale (FIES) questionnaire as per the Food and Agricultural Organization (FAO) guidelines. Our outcome variable was food insecurity, categorized as: 1) food secure (FIES score = 0–3), moderately food insecure (FIES score = 4–6), and severely food insecure (FIES score = 7–8). We did descriptive and multinomial regressions to analyze data using Stata version 16.

**Results:**

Between 2014 and 2018, the percentage of severe food insecurity fluctuated—42.81% in 2014, 37.79% in 2015, 38.98% in 2016, 45.41% in 2017, and 33.84% in 2018. Whereas that of moderate food insecurity increased from 23.55% to 27.33% except for 2016 and 2017 where the percentage increased to 32.33% and 27.46% respectively. In the logistic regression analysis, we found that respondents with lower than elementary education had a higher relative risk ratio of moderate (RRR = 1.45,95%CI = 1.22–1.72) and severe (RRR = 1.72, 95%CI = 1.46–2.02) food insecurity compared to those with secondary and higher education. Rural respondents had higher RRR of severe food insecurity (RRR = 1.37, 95%CI = 1.16–1.62) compared to those who lived in the urban areas. Compared with those in the richest wealth quintile, respondents in the poorest wealth quintile had 2.21 times higher RRR of moderate (RRR = 2.21, 95%CI = 1.69–2.87) and 3.58 times higher RRR of severe (RRR = 3.58, 95%CI = 2.81–4.55) food insecurity.

**Conclusion:**

About two-thirds of participants experienced some level of food insecurity in 2018. Lower levels of education, rural residency and poorer household wealth index areas were associated with a higher risk of food insecurity. National food security programs should focus on promoting education and improving socioeconomic condition of people especially in rural areas.

## Introduction

Food insecurity is assessed by four main elements, namely availability, accessibility, utilization and stability [1, 2] and can be categorized into mild-to-moderate and severe [3]. Numerous studies indicate that food insecurity is a pressing issue worldwide and characterized by inadequate access to safe and nutritionally adequate food due to various demographic, social and financial issues [4, 5]. Food insecurity is associated with a range of negative health outcomes and poor wellbeing [6, 7]. Previous studies suggest that food insecurity contributes to poor mental health conditions such as depression, stress and anxiety, suicidal ideation [8, 9], cardiovascular diseases [10], hypertension [11], and diabetes [12]. It’s also been found to be associated with double burden of malnutrition [13, 14] especially in low-income countries.

The 2022 Global Hunger Index estimates that hunger has increased significantly during last 10 years with over 800 million people affected in 2021, with the situation being far worse in the developing regions such as sub-Saharan Africa and South Asia [15, 16]. West and Central Africa have been particularly affected, with about 40 million people experiencing food and nutrition crises in 2022 [17]. Togo, being a country affected by widespread poverty, unemployment and malnutrition, faces serious challenges to ensure food security. The situation is furthermore aggravated by low agricultural productivity, population growth, land and environmental degradation, susceptibility to climate change [18, 19], and armed conflict [20, 21]. Due to prolonged terrorism activities, a significant number of the population of northern Togo have been displaced among and within countries in search of safety, exposing them to food insecurity [22]. According to the 2022 Cadre Harmonisé (CH) report, more than 386,000 Togolese, or 6.6 percent of the population, are food insecure, an increase of 89 percent compared to the previous year. Furthermore, approximately 1.1 million people, or 20 percent of the population, are at risk of falling into food insecurity, an 18 percent increase from the previous year [23].

In recent years, many government-led programs and international institutions have been investing on national food security to promote access to adequate and nutritious food across the country. Nonetheless, a large proportion of the population continue to suffer from food insecurity and malnutrition, with the situation being more prominent in the socioeconomically vulnerable groups. Studies have also shown that wealth inequality [24], gender inequality in access to resources [25, 26], lack of education [27, 28], large family size [29], place of residence [30], and age-groups particularly children before the age of five and elder people [31, 32] are among the sociodemographic factors that contribute to food insecurity. While there is a growing body of research that explore the factors associated with food insecurity and malnutrition at individual, household, and national levels, the majority of these studies have been conducted among children and older people in low- and middle-income countries [13, 33]. Despite the fact that food insecurity and malnutrition remain major public health concerns in Togo, few studies have specifically explored food insecurity at the individual level with regard to socio-economic factors. In light of this gap, the present study aimed to explore food insecurity among men and women aged 15 years and older in Togo. By investigating the socio-economic factors that contribute to food insecurity, the findings of this research can be used by policymakers and practitioners to design effective food security policies and programs for adult people not only in Togo but also in other West African countries.

## Methodology

### Data source and sampling technique

Data for this survey was carried out in Togo, a small West African country that borders Benin to the east, Ghana to the west, Burkina-Faso to the north, and the Gulf of Guinea to the south. As of April 21, 2023, the population of Togo was estimated to be nearly 9 million, compared to 8.6 million in 2022, with a land area of 56,785 sq km (21,925 sq mi) [23, 34]. According to the World Bank’s report in 2022, over half of the population lives in rural areas, survives on less than $1.90 a day, and relies on subsistence agriculture as their primary source of income [35]. Togo faces various social inequality challenges, compounded by low levels of education and healthcare, which have contributed to high levels of poverty especially in the rural areas. The United Nations’ Sustainable Development Goals (SDG) classification ranked Togo 143 out of 165 countries in 2021, with a particularly poor performance in SDG 1 (Poverty), SDG 5 (Gender), and SDG 10 (Fight against Inequalities) [36]. Despite some progress made in the last five years, Togo remains a low-income food-deficit country and is still categorized as a least developed country with a serious food insecurity situation [37].

In this cross-sectional study, we used data from the five waves (2014 to 2018) of the Gallup World Poll (GWP) for Togo. Since 2005, each year, the GWP, supported by FAO, has conducted annual uniform cross-country surveys in over 150 countries. The GWP collects information on attitudes and behaviours across various topics, including general well-being, demographic characteristics, educational level, food access, family economics, and employment [4, 38]. A total of 1,000 individuals in each country, aged 15 or older, were surveyed using randomly selected, nationally representative samples. The GWP uses a multi-stage sample technique that starts with categorizing countries according to their population sizes, randomly choosing families from each sampling unit, and then randomly choosing one person who is 15 or older from each home for an interview. Interviews are carried out by telephone in countries where more than 80% of the population have access to a phone and face-to-face everywhere else. In the case of Togo, date were collected using face-to-face interviews. Sampling weights are employed to guarantee that the final sample is representative of the full population aged 15 and over.

## Study variables

### Outcome variable: Measurement of food insecurity

For the first time in 2014, the Food and Agriculture Organization (FAO) through the Voices of the Hungry (VoH) project began collecting Food Insecurity Experience Scale (FIES) data which has been administered through the GWP [4]. The FIES measures the food insecurity experienced at the individual level based on a series of eight dichotomous questions, with “yes”/ “no” responses using the Rasch model [39]. The questions that are asked focus on self-reported experiences and behaviours related to food access. Respondents answer “yes” if they had been faced with increasing difficulties in accessing food because of lack of money or other resources, over the past 12 months, and “no” if otherwise (see Table 1) [4].

**Table 1:** The FIES questions.

N.	Short reference	Question wording: During the last 12 months, was there a time when, because of lack of money or other resources (Yes/No)
Q1	WORRIED	You were worried you would not have enough food to eat?
Q2	HEALTHY	You were unable to eat healthy and nutritious food?
Q3	FEWFOOD	You ate only a few kinds of foods?
Q4	SKIPPED	You had to skip a meal?
Q5	ATELESS	You ate less than you thought you should?
Q6	RUNOUT	Your household ran out of food?
Q7	HUNGRY	You were hungry but did not eat?
Q8	WHLDAY	You went without eating for a whole day?

For this study, the outcome variable was the food insecurity score based on the eight questions presented in *Table 1*. To describe the degree of food insecurity situation, the responses across the 8 items ranging from 0 to 8 were converted into aggregate scores and classified into 3 categories as follows: 1) food secure (raw score = 0–3), moderately food insecure (raw score = 4–6), and severely food insecure (raw score = 7–8).

### Explanatory variables

The explanatory variables included in this study are: year of survey (2014–2018), gender (male and female), age-groups (<19, 20–29, 30–39, 40–49, >49), educational status (Elementary or lower, secondary and higher), number of children in the household (0–2, and >2), number of adults in the household (1–2, and >2), place of residence (urban and rural), and wealth index (poorest, poorer, middle, rich and richest).

### Statistical analysis

All data analyses were done using Stata 16 version statistical software. Due to the clustered nature of the data, we used the survey command (svy) to specify the relevant characteristics of sampling design, including sampling weights, clustering, stratification and poststratification. To obtain sampling weights, post-stratification is used by GWP and ensured the adjusted weights for non-response and non-overlapping groups (strata) and correct bias from overrepresented and underrepresented samples. This aligns the sample to be representative and match the characteristics of the population. For descriptive analysis, we first, provided the questionnaire that describes the 8 questions on food insecurity situation in the last 12 months (Table 1); second, based on responses to the FIES of the surveys’ data, we calculated the scores of food insecurity and categorized them into three labels: 1) food secure, 2) moderately food insecure and 3) severely food insecure. Next, we calculated the frequency distribution of participants by type of food insecurity by survey year and other sociodemographic variables. Lastly, we used multinomial regression techniques to measure the association between food insecurity and the sociodemographic variables. In the first steps, bivariate logit regression was carried out to compute the relative risk ratio (RRR) of the association between food insecurity (outcome variable) and the explanatory variables. However, all variables used in the bivariate analysis were added to the multinomial logistic regression analysis in the second step. In this, we performed the analyses to calculate the RRR to quantify the association between food insecurity and sociodemographic characteristics, together with the 95% confidence intervals. We also tested for multicollinearity using the variance inflation factor (VIF) method, and the values for all covariates were < 10 (Mean  =  1.13, range: 1.06–3.30) which indicated no multicollinearity among the predictor variables. For all analysis, a p-value of <0.05 was considered statistically significant.

### Ethical clearance

Additional ethical approval was not required for this study as the data were secondary and are available in the public domain in an anonymous form.

## Results

Table 2 presents the percentage distribution of food insecurity across sociodemographic characteristics of respondents. In total 4754 participants aged above 15 years were included in this study. The percentage of participants with moderate food insecurity (32.33%) was highest in 2016 and that of severe food security (45.41%) was the highest in 2017. The percentage of moderate food insecurity was the lowest in 2015 (27.07%) and that of severe food insecurity 2018 (33.84%). In terms of gender, the percentage of severe food insecurity was higher than moderate food insecurity in both men (40.73%) and women (38.69%). Participants aged between 20–29 years had a higher percentage of moderate food insecurity (29.75%), while those aged 40–49 years had higher percentage of severe food insecurity (43.09%). It also showed that food insecurity decreases with the level of education. The percentage of moderate (27.81%) and severe (46.27%) food insecurity was relatively higher among respondents with lower than elementary education than those with secondary and higher education. Regarding areas of residence, respondents who lived in rural areas had higher percentage of moderate (27.59%) and severe (42.49%) food insecurity than those who lived in urban areas. The percentage of moderate (29.01%) and severe (44.23%) food insecurity was relatively higher in the households with >2 children. Also, the percentage of moderate food insecurity (27.75%) was higher in households whith 1–2 adults, while that of severe food insecurity (39.91%) was the highest in households with >2 adults. As for the wealth index, the percentage of moderate (poorest 26.42% vs richest 24.19%) and severe (poorest 50.81% vs richest 27.20%) food insecurity was relatively higher for the poorest households compared with the richest.

**Table 2:** Sociodemographic characteristics of respondents (n = 4754).

Variables	FS	MFI	SFI	P-value
	N (%)	N (%)	N (%)	
*Year*				<0.001***
2014	330 (33.64)	231 (23.55)	420 (42.81)	
2015	331 (35.14)	255 (27.07)	356 (37.79)	
2016	276 (28.69)	311 (32.33)	375 (38.98)	
2017	257 (27.14)	260 (27.46)	430 (45.41)	
2018	358 (38.83)	252 (27.33)	312 (33.84)	
*Gender*				0.092
Male	826 (31.32)	737 (27.95)	1074 (40.73)	
Female	726 (34.29)	572 (27.02)	819 (38.69)	
*Age-groups*				0.013*
<19	276 (36.32)	180 (23.68)	304 (40.00)	
20–29	549 (33.13)	493 (29.75)	615 (37.12)	
30–39	329 (30.98)	302 (28.44)	431 (40.58)	
40–49	188 (30.23)	166 (26.69)	268 (43.09)	
>49	210 (32.16)	168 (25.73)	275 (42.11)	
*Education*				<0.001***
Elementary or lower	591 (25.92)	634 (27.81)	1055 (46.27)	
Secondary and High	961 (38.84)	675 (27.28)	838 (33.87)	
*Place of residence*				<0.001***
Urban	498 (40.45)	337 (27.38)	396 (32.17)	
Rural	1054 (29.92)	972 (27.59)	1497 (42.49)	
*Number of Children in HH*				<0.001***
0–2	1100 (35.89)	819 (26.72)	1146 (37.39)	
>2	452 (26.76)	490 (29.01)	747 (44.23)	
*Number of adults in HH*				0.924
1–2	883 (32.50)	754 (27.75)	1080 (39.75)	
>2	669 (32.84)	555 (27.25)	813 (39.91)	
*Wealth index*				<0.001***
Poorest	169 (22.78)	196 (26.42)	377 (50.81)	
Poorer	163 (21.01)	212 (27.32)	401 (51.68)	
Middle	242 (27.59)	258 (29.42)	377 (42.99)	
Richer	333 (32.27)	322 (31.20)	377 (36.53)	
Richest	645 (48.61)	321 (24.19)	361 (27.20)	

FS = Food Security; MFI = Moderate Food Insecurity; SFI = Severe Food Insecurity HH = Household

* *p* < 0.05

** *p* < 0.01

*** *p* < 0.001

### Factors associated with household food insecurity in Togo

*Tables 3 and 4* show the factors associated with household food insecurity. Supplementary tables (S1–S5 Files) with the same analyses for each year (2014, 2015, 2016, 2017 and 2018 respectively) have also been provided.Table *3* presents the results of a bivariate logistic regression model in which all variables added to the multinomial logit regression analysis. Table 4 shows the results of the multinomial logit regression analysis on the factors associated with household food insecurity.

**Table 3:** Bivariate logit regression model for the factors associated with household food insecurity.

Variables	MFI vs FS		SFI vs FS	
	RRR, 95%CI	P-value	RRR, 95%CI	P-value
*Year* (2014)	1		1	
2015	1.10 (0.87–1.39)	0.423	0.85 (0.69–1.04)	0.112
2016	1.61 (1.27–2.03)	<0.001***	1.07 (0.86–1.32)	0.546
2017	1.45 (1.14–1.84)	0.003**	1.31 (1.06–1.62)	0.011*
2018	1.01 (0.80–1.27)	0.963	0.68 (0.56–0.84)	<0.001***
*Gender* (Male)	1		1	
Female	0.88 (0.76–1.02)	0.099	0.87 (0.76–0.99)	0.039*
Age-groups (<19)	1		1	
20–29	1.38 (1.10–1.72)	0.005**	1.02 (0.83–1.24)	0.868
30–39	1.41 (1.10–1.80)	0.006**	1.19 (0.96–1.48)	0.117
40–49	1.35 (1.02–1.79)	0.034*	1.29 (1.01–1.66)	0.041*
>49	1.23 (0.93–1.62)	0.147	1.19 (0.93–1.52)	0.162
*Education* (Secondary/high)	1		1	
Elementary or lower	1.53 (1.32–1.77)	<0.001***	2.05 (1.79–2.35)	<0.001***
*Place of residence* (Urban)	1		1	
Rural	1.36 (1.16–1.60)	<0.001***	1.79 (1.53–2.08)	<0.001***
*Children in HH* (0–2)	1		1	
>2	1.46 (1.24–1.70)	<0.001***	1.59 (1.37–1.83)	<0.001***
*Number of adults in HH* (>2)	1		1	
1–2	1.03 (0.89–1.19)	0.703	1.01 (0.88–1.15)	0.926
*Wealth index* (Richest)	1		1	
Richer	1.94 (1.59–2.38)	<0.001***	2.02 (1.66–2.46)	<0.001***
Middle	2.14 (1.72–2.67)	<0.001***	2.78 (2.26–3.42)	<0.001***
Poorer	2.61 (2.05–3.34)	<0.001***	4.40 (3.52–5.49)	<0.001***
Poorest	2.33 (1.82–2.98)	<0.001***	3.99 (3.19–4.98)	<0.001***

FS = Food Security; MFI = Moderate Food Insecurity; SFI = Severe Food Insecurity; HH = Household

RRR; 95% confidence intervals in brackets

* *p* < 0.05

** *p* < 0.01

*** *p* < 0.001

**Table 4:** Multinomial logit regression model for the factors associated with household food insecurity.

Variables	MFI vs FS		SFI vs FS	
	RRR, 95%CI	P-value	RRR, 95%CI	P-value
*Year* (2014)	1		1	
2015	1.07 (0.84–1.36)	0.580	0.85 (0.68–1.05)	0.132
2016	1.66 (1.31–2.11)	<0.001***	1.17 (0.93–1.46)	0.174
2017	1.51 (1.17–1.94)	0.001***	1.46 (1.16–1.83)	0.001***
2018	1.08 (0.85–1.38)	0.526	0.80 (0.64–0.99)	0.048*
Age-groups (<19)	1		1	
20–29	1.51 (1.20–1.91)	0.001***	1.19 (0.96–1.48)	0.104
30–39	1.37 (1.06–1.77)	0.017*	1.17 (0.93–1.48)	0.182
40–49	1.26 (0.94–1.69)	0.122	1.18 (0.90–1.53)	0.226
>49	1.09 (0.82–1.46)	0.237	0.99 (0.76–1.29)	0.942
Gender (Male)	1		1	
Female	0.79 (0.67–0.92)	0.003**	0.72 (0.62–0.84)	<0.001***
Education (Secondary/higher)	1		1	
Elementary or lower	1.45 (1.22–1.72)	<0.001***	1.72 (1.46–2.02)	<0.001***
*Place of residence* (Urban)	1		1	
Rural	1.17 (0.98–1.39)	0.077	1.37 (1.16–1.62)	<0.001***
*Children in HH* (0–2)	1		1	
>2	1.11 (0.94–1.33)	0.226	1.06 (0.90–1.24)	0.515
Number of adults in HH (>2)	1		1	
1–2	110 (0.93–1.30)	0.267	1.18 (1.01–1.37)	0.038*
*Wealth index* (Richest)	1		1	
Richer	1.89 (1.53–2.33)	<0.001***	1.93 (1.58–2.37)	<0.001***
Middle	2.09 (1.65–2.64)	<0.001***	2.62 (2.10–3.27)	<0.001***
Poorer	2.43 (1.87–3.17)	<0.001***	3.92 (3.07–5.00)	<0.001***
Poorest	2.21 (1.69–2.87)	<0.001***	3.58 (2.81–4.55)	<0.001***

FS = Food Security; MFI = Moderate Food Insecurity; SFI = Severe Food Insecurity; HH = Household

RRR; 95% confidence intervals in brackets

* *p* < 0.05

** *p* < 0.01

*** *p* < 0.001

In 2016, compared with 2014, the relative risk ratio of moderate food insecurity had increased by 66% (RRR = 1.66, 95%CI = 1.31–2.11). Similarly for 2017, compared with 2014, the relative risk ratio of moderate food insecurity had increased by 51% (RRR = 1.51, 95%CI = 1.17–1.94) and that of severe food insecurity by 46% (RRR = 1.46, 95%CI = 1.16–1,83).

We also found that the relative risk ratio of moderate food insecurity had increased by 51% and 37% among respondents aged between 20–29 years and 30–39 years (RRR = 1.51, 95%CI: 1.20–1.91) and (RRR = 1.37, 95%CI = 1.06–1.77) respectively, compared to those of aged under 19 years. For severe food insecurity, no significant association was found with age-group.

Having lower than elementary education showed a higher relative risk ratio of moderate (RRR = 1.45,95%CI = 1.22–1.72) and severe (RRR = 1.72, 95%CI = 1.46–2.02) food insecurity compared to those with secondary and higher education.

Regarding place of residency, respondents who lived in the rural areas had higher relative risk ratio of severe food insecurity (RRR = 1.37, 95%CI = 1.16–1.62) compared to those who lived in the urban areas. The association between place of residence and moderate food insecurity was not significant.

Also, having 1 or 2 adults in a household, had higher relative risk ratio of severe food insecurity (RRR = 1.18, 95%CI = 1.01–1.37) compared to those with >2 adults in a household. For moderate food insecurity, we did not find any significant result with number of adults in households.

In terms of wealth index, all respondents from the first four wealth quintiles had higher relative risk ratio of moderate and severe food insecurity than the fifth (richest) wealth quintile. For instance, respondents who were from the poorest wealth quintile had 2.21 times and 3.58 times higher relative risk ratio of moderate (RRR = 2.21, 95%CI = 1.69–2.87) and severe (RRR = 3.58, 95%CI = 2.81–4.55) food insecurity respectively compared with those in the richest wealth category. Also, the relative risk ratio of moderate and severe food insecurity was 2.43 times (95% CI = 1.87–3.17) and 3.92 times (95%CI = 3.07–5.00) higher for respondents in the poorer wealth quintile compared with those in the richest quintile.

However, regarding gender difference, our results also showed that women had relatively lower risk ratio of moderate and severe food insecurity (RRR = 0.79, 95%CI = 0.67–0.92 and RRR = 0.72, 95%CI = 0.62–0.84) respectively compared with men.

## Discussion

This study aimed to explore food insecurity at the individual level based on socio-economic factors among men and women 15 years and older in Togo. We calculated relative risk ratios of being moderately and severely food insecured by using data from the Togo Gallup World Poll (GWP), supported by FAO. The findings of the present study provide insights on the proportion of food insecurity across the sociodemographic characteristics of the respondents. The results indicate that between 2014 and 2018, the proportion of moderate food insecurity was lower [40] but that of severe food insecurity was higher than previous studies [41, 42]. This difference may be due to poor harvest, poor soil conditions, flood, drought, climate change, poor economic growth, high household size, unemployment, food price shock, poor food security policies, or other [43–45].

We also found in our regression model that food insecurity decreases with the level of education. Respondents who had lower than elementary education had a higher relative risk ratio of moderate and severe food insecurity compared to those with higher education. This finding reinforces evidence from previous studies done in the Congo Basin [46], Pakistan [47], and Ghana [48, 49] where the level of education is negatively associated with the rate of food insecurity. Similar finding was also obtained by Mutisya and colleagues in their longitudinal study done in Kenya where food insecurity decreased with higher level of education [28]. This could be explained by the fact that better education linked to better income-earning opportunities and greater knowledge of healthy and nutritious foods, can lead to better food choices and higher capacity to avoid food shortages.

The results also highlight the impact of age, gender, and area of residence on food insecurity. Specifically, respondents aged between 20–29 years and 30–39 years had a higher relative risk ratio of moderate food insecurity compared to those aged under 19 years. This finding is in agreement with earlier works in which young adults were disproportionately affected by moderate or severe food insecurity challenges [50, 51]. The possible rational might be due to the fact that respondents of these age-groups were unemployed or paid less and may have limited financial resources to access food compared to those aged under19 years who may dependent entirely on their parents for provision of food and other needs.

Regarding gender difference, it should be noted that women had comparatively lower relative risk ratio of moderate and severe food insecurity than men. This finding is consistent with the work of Gnedeka and Wonyra in Togo [40] but inconsistent with the study done by Broussard in 146 countries around the world where women have a higher probability of being food insecure relative to men [52]. This may be due to the sociocultural differences between the two countries. In Togo, like in the other Western African countries, the household tasks including buying and preparing food is generally performed by women. Men, on the other hand, are involved in income-generation activities and hence are more aware and concerned about the financial difficulties that may affect the affordability of food in near future. In addition, women are also supported through various social benefit programs by the government which can reduce the financial burden to some extent and help promote food security [45].

In this study, geographical disparities were also found to be a contributing factor for food insecurity. Respondents in rural areas had a higher relative risk ratio of severe food insecurity compared to those who lived in urban areas. This finding adds to the growing body of literature indicating that rural residents had higher risk of being food insecured than urban residents [47, 53]. This is perhaps because there is inadequate access to affordable food in the rural areas, as well as limited infrastructure like public transportation, supermarkets, and stores where a variety of food items can be found [30, 54]. In addition to this, the frequent flood and drought further impact food security, especially in the rural areas [55]. Although residents in the rural areas are usually involved in agricultural activities, they rely on the revenue from their produce to purchase non-agricultural foods and services such as healthcare and education. Thus, limited sources of income during the off seasons can put them at higher risk of food insecurity.

Also, the number of adults in households influenced food insecurity. We found in this study that having 1 or 2 adults in a household had higher relative risk ratio of severe food insecurity compared to those with >2 adults in a household. This is possibly because household with higher number of adults might have higher income opportunities which contributes to higher affordability of food items.

Additionally, socio-economic inequalities and socio-cultural factors may also be associated with food insecurity. Findings from this study showed that respondents who were from the lower household wealth quintiles had significantly higher relative risk ratio of moderate and severe food insecurity than those from the richest households. This finding is similar to previous cross-sectional studies in which people in the poorest wealth quintile were more likely to experience moderate or severe food insecurity compared with those in the richest wealth category [56, 57]. Similar results were found by a cohort study done in India by Rautela and colleagues that the risk of being insecure increased with poorest household quintiles compared to those with the wealthiest household quintiles [58]. Respondents who were from wealthy household quintiles might have higher financial means or access to resources to get food than those from poorest households. This may further be attributed to the lower capacity to purchase sufficient food for the household. This indicates that wealth inequality is a major factor contributing to food insecurity and vice versa. Regarding the socio-cultural factors, there are many ways they can impact food insecurity. For instance, in religious context in Togo, many indigenous communities prefer waiting for the new harvest to be offered to their gods before consuming them, which can impact the level of food consumption even when there is enough food to eat. Also, farmers are not authorised to sell and consumers are not authorised to purchase certain products (such as yams and beans) before reaching the time of offering. Future qualitative studies should explore the role of sociocultural factors in greater details.

### Strengths and limitations

This study presents some major strengths. Firstly, to the best of our knowledge, this study is one of the first survey that explored the factors associated with food insecurity in Togo using data from the five waves (2014 to 2018) of the Gallup World Poll (GWP). Secondly, face-to-face interviews were used to randomly select respondents from households that integrated a large number of socioeconomic, wealth index, education and geographical disparities as explanatory variables that are associated with food insecurity. Finally, the sample size of this study was relatively large and nationally representative. As limitations, our analysis relied on self-reported data, which may have introduced measurement bias and underreporting of sensitive issues such as food insecurity. Moreover, we were unable to assess the quality and quantity of food consumed by the participants, which could have provided more insights into the mechanisms underlying the association between food insecurity and education, wealth index, and geographical disparities. Another limitation of our study was the inability to include other relevant variables that could influence food insecurity, such as employment status, household size, and health status. In addition, we did not explore the potential effect of cultural practices and beliefs on food insecurity, which could vary across different regions in Togo. Lastly, although our study provided evidence on the association between education, wealth index, and geographical disparities with food insecurity, we were unable to examine the potential interactions between these variables and their combined effect on food insecurity. Future research should aim to address these limitations by employing longitudinal study designs, incorporating objective measures of food intake, and including a wider range of relevant variables.

## Conclusion

This study provides valuable insights into the percentage and factors of food insecurity in Togo between 2014 and 2018. The results demonstrate that food insecurity is a significant problem affecting a substantial portion of the population, particularly among those who are less educated, living in rural areas, having 1 or 2 adults in a household and being in the poorest wealth quintile households. These findings suggest that interventions aimed at improving education, increasing access to affordable and healthy food in rural areas, and reducing wealth inequalities may help address the issue of food insecurity in Togo. It is important for policymakers and practitioners to take into account these socio-economic factors when designing and implementing targeted interventions to alleviate food insecurity in the country. Overall, this study contributes to the growing body of knowledge on food insecurity and provides insights that can inform policy and practice aimed at improving food security for the most vulnerable populations in Togo. Based on these findings, it is recommended that food security programs in Togo invest on education, women empowerment, job creation, and reduce geographic inequalities.

## Supporting information

S1 File Bivariate and multinomial logit regression model for the factors associated with household food insecurity in Togo in 2014. (PDF)

S2 File Bivariate and multinomial logit regression model for the factors associated with household food insecurity in Togo in 2015. (PDF)

S3 File Bivariate and multinomial logit regression model for the factors associated with household food insecurity in Togo in 2016. (PDF)

S4 File Bivariate and multinomial logit regression model for the factors associated with household food insecurity in Togo in 2017. (PDF)

S5 File Bivariate and multinomial logit regression model for the factors associated with household food insecurity in Togo in 2018. (PDF)

## Abbreviations

CH: Cadre Harmonise
CI: Confidence Interval
FAO: Food and Agricultural Organization
FIES: Food Insecurity Experience Scale
FS: Food Security
GWP: Gallup World Poll
MFI: Moderate Food Insecurity
RRR: Relative Risk Ratio
SFI: Severe Food Insecurity
VoH: Voices of the Hungry
