# Supplementary material for: Examining the disparities: A cross-sectional study of socio-economic factors and food insecurity in Togo
Source: PLoS One. 2023 Nov 27;18(11):e0294527. doi: 10.1371/journal.pone.0294527 (PMC10681261; doi:10.1371/journal.pone.0294527)
Supplement: S1 File — (PDF) [file pone.0294527.s001.pdf]

Bivariate and Multinomial logit regression model for the factors associated with household food insecurity in Togo in 2014

	Bivariate logit regression		Multinomial logit regression	
Variables	MFI vs FS	SFI vs FS	MFI vs FS	SFI vs FS
	RRR, 95%CI	RRR, 95%CI	RRR, 95%CI	RRR, 95%CI
**Year 2014**				
Age-groups (<19)	1	1	1	1
20-29	1.16 [0.69-1.95]	0.97 [0.62-1.51]	1.24 [0.73-2.12]	1.09 [0.68-1.75]
30-39	1.03 [0.59-1.79]	1.14 [0.71-1.81]	1.03 [0.57-1.86]	1.16 [0.70-1.93]
40-49	1.31 [0.73-2.37]	1.08 [0.65-1.80]	1.21 [0.65-2.26]	0.94 [0.54-1.64]
>49	0.90 [0.51-1.62]	0.88 [0.54-1.43]	0.86 [0.46-1.60]	0.73 [0.43-1.26]
Gender (Male)	1	1	1	1
Female	1.34 [0.96-1.88]	1.30 [0.97-1.73]	1.24 [0.87 -1.78]	1.09 [0.80-1.50]
Education (Secondary/high)	1	1	1	1
Elementary or lower	1.52* [1.08-2.14]	2.04*** [1.80,3.30]	1.36 [0.92-2.02]	2.07*** [1.46-2.94]
Place of residence (Urban)	1	1	1	1
Rural	1.39 [0.94-2.07]	2.29*** [1.59-3.31]	1.16 [0.77-1.77]	1.66* [1.12-2.45]
Number of Children (0-2)	1	1	1	1
>2	1.61* [1.11-2.34]	1.64** [1.19-2.28]	1.23 [0.81-1.87]	0.98 [0.67-1.42]
Number of adults in HH (>2)	1	1	1	1
1-2	0.79 [0.56-1.11]	0.71* [0.53-0.94]	1.03 [0.71-1.50]	0.99 [0.71-1.38]
Wealth index (Richest)	1	1	1	1
Richer	2.44*** [1.51-3.94]	2.82*** [1.82-4.38]	2.26*** [1.37-3.73]	2.48*** [1.57-3.93]
Middle	2.45*** [1.48-4.07]	3.70*** [2.36-5.80]	2.11** [1.21-3.66]	3.02*** [1.84-4.94]
Poorer	2.88*** [1.63-5.10]	5.86*** [3.57-9.62]	2.33** [1.23-4.41]	4.46*** [2.56-7.78]
Poorest	1.40 [0.82-2.37]	2.55*** [1.63-3.98]	1.23 [0.70-2.14]	2.06** [1.28-3.30]

FS = Food Security; MFI = Moderate Food Insecurity; SFI= Severe Food Insecurity; HH= Household

RRR; 95% confidence intervals in brackets

* $p < 0.05$, ** $p < 0.01$, *** $p < 0.001$
